# Overtreatment by hysterectomy for cervical intraepithelial neoplasia: Medico-legal aspects

**DOI:** 10.18502/ijrm.v21i6.13639

**Published:** 2023-07-24

**Authors:** Camilla Cecannecchia, Fernanda Cioffi, Andrea Cioffi

**Affiliations:** ^1^Department of Anatomical, Histological, Forensic and Orthopaedic Sciences, Sapienza University of Rome, Italy.; ^2^Fertilitas Day Surgery Reproductive Medicine, Salerno, Italy.; ^3^Department of Clinical and Experimental Medicine, Section of Legal Medicine, University of Foggia, Italy.

##  Dear Editor,

Recent scientific evidence shows that the number of hysterectomies is far greater than those needed. In the last 10 yr in Italy (and not only), the number of hysterectomies performed for cervical intraepithelial neoplasia (CIN) has also increased in a worrying way (1). However, the recommendations of the World Health Organization (2) and the most recent European guidelines (3) make this clear that in the case of CIN 1 of a histological high-grade squamous intraepithelial lesion (i.e., CIN 2-3) not exceeding the basal membrane, and of invasive cervical cancer up to stage T1a1, conservative diagnostic-therapeutic management should be preferred. In the above cases, hysterectomy constitutes an overtreatment as the risk of complications of surgery outweighs any health benefit to the patient. In some circumstances, hysterectomy can be a (partially or totally) resolving tool for CIN: microinvasion, histologically confirmed recurrent high-grade CIN, co-presence of other uterus pathologies that worsen the overall clinical-gynecological picture, such as prolapse, fibroids, pelvic inflammatory disease, and endometriosis (4). Except in these cases, less invasive methods for the treatment of CIN 1 and of histological high-grade squamous intraepithelial lesion are recommended.

The reasons behind the inadequate use of hysterectomy (i.e., in the absence of specific indications) are attributable both to the insufficient scientific updating of some gynecologists, and to the spread of the phenomenon of defensive medicine, also called defensive medical decision-making (5). The actions of the gynecologist are no longer guided by scientific evidence but motivated primarily by litigious concerns. The increased incidence of medico-legal litigation in the healthcare sector inevitably instils in the surgeons' minds the fear that their decisions may be challenged. Namely, gynecologists end up practicing more than the necessary hysterectomies (even in the absence of specific indications) in fear that using less invasive procedures may be challenging in case of an unlikely progression of the neoplasm.

The increase in unnecessary hysterectomies has significant medical-legal repercussions. First, a hysterectomy is an invasive surgery associated with a risk of mortality of 0.4% and a much higher risk of intra- and postoperative complications (1). Complications and harms of an unnecessary hysterectomy can expose the doctor and/or healthcare facility to a greater risk of legal defeat. In fact, both will be called to justify the use of an avoidable invasive procedure. In addition, a hysterectomy can also cause mental damage in women (6).

The uterus preservation can help to preserve fertility, and that is why, in primis, this issue has been discussed here, but uterus is not an organ exclusively aimed at procreation but is, in all respects, an indispensable part of the sexual identity of the woman, regardless of age. Therefore, it is urgent to implement the quality of the scientific update of gynecologists; it is also essential to carry out a standardized control of healthcare facilities in order to assess the adequacy of hysterectomies. Such strategies are crucial to protect gynecologists from the false sense of safety of defensive medicine and to put the patient at the center of a treatment decision.
